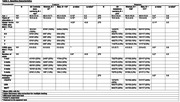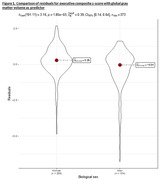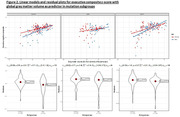# Influence of biological sex on early cognitive performance in FTLD mutation carriers: an ALLFTD study

**DOI:** 10.1002/alz.088312

**Published:** 2025-01-09

**Authors:** Jesús Garcia Castro, Sara Rubio‐Guerra, Judit Selma González, Molly B Memel, Oriol Dols‐Icardo, Alexandre Bejanin, Olivia Belbin, Juan Fortea, Daniel Alcolea, Maria Carmona‐Iragui, Isabel Barroeta, Miguel A Santos‐Santos, Mª Belen Sánchez‐Saudinós, Isabel Sala, Hilary W. Heuer, Adam M. Staffaroni, Kaitlin B. Casaletto, Brad F. Boeve, Adam L. Boxer, Howard J. Rosen, Alberto Lleo, Ignacio Illán‐Gala

**Affiliations:** ^1^ Hospital de la Santa Creu i Sant Pau, Barcelona, Barcelona Spain; ^2^ University of San Francisco, San Francisco, CA USA; ^3^ University of California, San Francisco, San Francisco, CA USA; ^4^ Mayo Clinic, Rochester, MN USA

## Abstract

**Background:**

Accumulating evidence indicates that biological sex may influence clinical manifestation within the spectrum of frontotemporal lobar degeneration (FTLD), implying differences in cognitive reserve. Nonetheless, investigations into the impact of biological sex during the preclinical and minimally symptomatic stages of FTLD are lacking.

**Method:**

We included 275 mutation carriers (158 females; 127 with C9orf72, 68 with GRN, and 80 with MAPT mutations) and 161 non‐carrier familial controls (97 females) from the ALLFTD Consortium (Staffaroni et al., Nat Medicine 2022). Participants underwent magnetic resonance imaging (MRI; 348 baseline, 338 longitudinal) and neuropsychological evaluations (394 baseline, 507 longitudinal). Behavioral symptoms were characterized with the Revised Self‐Monitoring Scale (RSMS) and the Neuropsychiatric Inventory (NPI severity scores). MRI‐derived regional volume estimates (RVE) were computed. Cognitive measures and RVE were normalized against sex‐matched controls. Cognitive composites (language, executive function, and visuospatial function) were calculated by averaging raw scores for each domain. Clinical characteristics and RVE comparisons were made between male and female participants. We adopted the residuals approach to explore behavioral and cognitive reserve by fitting a linear regression model for executive z‐scores as the response value and age, education, and RVE as explanatory variables.

**Result:**

In mutation carriers, sex was not a significant differentiator in age, education level, disease severity, or mutation frequency. Most mutation carriers (188, 68%) were asymptomatic or mildly symptomatic at baseline. However, female mutation carriers exhibited significantly lower visuospatial performance at baseline (Cohen's d = ‐0.34, 95% CI[‐0.58, ‐0.09], p=.001). This difference remained significant among asymptomatic GRN mutation carriers (Cohen’s d = ‐0.73, 95% CI[‐1.22, ‐0.23], p=0.003) but not in other mutations. Following the residuals approach, female mutation carriers showed higher executive performance than males for the same amount of both frontotemporal and global atrophy as quantified by RVE (Cohen's d=0.45, 95 % CI[0.22, 0.67], p<.001). The observed higher executive reserve in women was particularly pronounced in C9orf72 carriers (Cohen's d=0.77, 95% CI[0.35, 1.20], p<.001) but not significant in GRN (p=0.48) and MAPT carriers (p=0.07). No differences in behavioral reserve reached statistical significance.

**Conclusion:**

Female sex might modulate early cognitive performance and confer higher executive reserve in FTLD mutation carriers.